# The *l*_1_-*l*_2 _regularization framework unmasks the hypoxia signature hidden in the transcriptome of a set of heterogeneous neuroblastoma cell lines

**DOI:** 10.1186/1471-2164-10-474

**Published:** 2009-10-15

**Authors:** Paolo Fardin, Annalisa Barla, Sofia Mosci, Lorenzo Rosasco, Alessandro Verri, Luigi Varesio

**Affiliations:** 1 Laboratorio di Biologia Molecolare, Giannina Gaslini Institute, Largo G Gaslini 5, I-16147 Genova, Italy; 2 Dipartimento di Informatica e Scienze dell' Informazione, Università di Genova, via Dodecaneso 35, I-16146 Genova, Italy; 3 Dipartimento di Fisica, Università di Genova, via Dodecaneso 33, I-16146 Genova, Italy; 4 Center for Biological & Computational Learning, MIT, 43 Vassar Street, Cambridge, MA, USA

## Abstract

**Background:**

Gene expression signatures are clusters of genes discriminating different statuses of the cells and their definition is critical for understanding the molecular bases of diseases. The identification of a gene signature is complicated by the high dimensional nature of the data and by the genetic heterogeneity of the responding cells. The *l*_1_-*l*_2 _regularization is an embedded feature selection technique that fulfills all the desirable properties of a variable selection algorithm and has the potential to generate a specific signature even in biologically complex settings. We studied the application of this algorithm to detect the signature characterizing the transcriptional response of neuroblastoma tumor cell lines to hypoxia, a condition of low oxygen tension that occurs in the tumor microenvironment.

**Results:**

We determined the gene expression profile of 9 neuroblastoma cell lines cultured under normoxic and hypoxic conditions. We studied a heterogeneous set of neuroblastoma cell lines to mimic the in vivo situation and to test the robustness and validity of the *l*_1_-*l*_2 _regularization with double optimization. Analysis by hierarchical, spectral, and k-means clustering or supervised approach based on t-test analysis divided the cell lines on the bases of genetic differences. However, the disturbance of this strong transcriptional response completely masked the detection of the more subtle response to hypoxia. Different results were obtained when we applied the *l*_1_-*l*_2 _regularization framework. The algorithm distinguished the normoxic and hypoxic statuses defining signatures comprising 3 to 38 probesets, with a leave-one-out error of 17%. A consensus hypoxia signature was established setting the frequency score at 50% and the correlation parameter ε equal to 100. This signature is composed by 11 probesets representing 8 well characterized genes known to be modulated by hypoxia.

**Conclusion:**

We demonstrate that *l_1_-l*_2 _regularization outperforms more conventional approaches allowing the identification and definition of a gene expression signature under complex experimental conditions. The *l_1_-l*_2 _regularization and the cross validation generates an unbiased and objective output with a low classification error. We feel that the application of this algorithm to tumor biology will be instrumental to analyze gene expression signatures hidden in the transcriptome that, like hypoxia, may be major determinant of the course of the disease.

## Background

Clues to the prognosis of cancer are reflected at the time of surgical removal in the pattern of gene expression in the primary tumor. The ultimate goal is to identify specific "gene expression signatures" that define subsets of tumors and that will ultimately allow to predict the clinical course. Unsupervised analysis of the gene expression pattern has led to the definition of "gene expression signatures" that add independent prognostic information to that provided by a risk assessment based solely on clinical-pathologic factors. One limitation of the unsupervised cluster analysis is the lack of appreciation of the tumor pathology, which makes these signatures difficult to interpret with respect to the underlying cancer biology which comprises the intrinsic properties of the cancer cell, such as activation of transforming genes, and the response to signals generated within the tissue microenvironment, such as the hypoxic situation occurring in poorly vascularized or necrotic areas of the tumor. Ultimately, finding gene signatures that can be linked to the molecular mechanisms of cancer development is critical for translating these markers into the clinic. Alternative strategies to combine the prognostic value and biologic knowledge are being developed. Specifically, gene expression signatures are derived from in vitro studies on the pathophysiology of the disease. This is a novel approach standing on the concept that the tumor biology will give us the clues to characterize the outcome of the disease.

In this manuscript, we address the above-mentioned issues by developing a novel approach to identify the signature of low oxygen tension (hypoxia) in a set of neuroblastoma cell lines. Oxygen is essential for aerobic metabolism in all mammalian cells. To maintain function and homeostasis, cells have to be able to sense and respond to inadequate oxygen levels. The O_2 _levels within the neoplastic lesion are an important factor in determining the tumor phenotype [1] and hypoxia is associated with metastatic spread, resistance to radio- and chemotherapy and poor prognosis [1-3]. The cellular response to hypoxia is caused by changes in gene expression [4-6] through the activation of several transcription factors among which the hypoxia-inducible transcription factor-1α (HIF-1α) [1,7], and -2α (HIF-2α) [8] are those taken as indicators of a hypoxic status of the cell. HIFs transactivate the hypoxia-responsive element (HRE) present in the promoter or enhancer elements of many genes encoding angiogenic, metabolic and metastatic factors [3,9,10]. Although hypoxia responses are thought to be evolutionarily conserved in all mammalian cells [11,12] not every cell responds to hypoxia in an identical fashion. Although certain biochemical pathways are common hypoxia targets, the specific genes modulated by hypoxia within each pathways will depend heavily on the nature, type and genetic makeup of the responding cell [5,6,13]. In other words, hypoxia-induced common biochemical pathways may utilize different genes depending on the cell type.

Neuroblastoma is the most common pediatric solid tumor, deriving from immature or precursor cells of the ganglionic lineage of the sympathetic nervous system (SNS) [14,15]. Neuroblastoma shows notable heterogeneity, with regard to both histology and clinical behavior [16]. The outcome of the disease ranges from rapid progression and poor clinical outcome, to spontaneous regression into benign ganglioneuroma [17]. The heterogeneity of neuroblastoma (NB) cells is found also in the cell lines derived from the fresh tumors which manifest various degree of differentiation and chromosomal alteration. For example, the amplification and/or expression of MYCN oncogene is a relatively frequent event, that is indicative of poor prognosis in fresh tumors and is present in several cell lines which share an aggressive behavior [18]. Recent data of microarray analysis confirm the existence of different patterns of gene expression profile among different NB cell lines [13]. The heterogeneity of the NB cell transcriptome complicates the identification of specific gene expression signatures associated to defined biological responses such as environmental stimulation that, albeit biologically very important, may be overshadowed by major genetic alterations as those caused by oncogenes which impact on several aspects of cell physiology. This problem is of major concern when several different NB cell lines have to be compared in in vitro studies.

The problem of identifying a gene signature, namely a significant group of variables, is aggravated by the typical high dimensional nature of the data. Complexity grows even more when the heterogeneity of the cells must be factored in. Several feature selection techniques have been proposed to deal with these problems (for review see [19]). The number of data available for a single study is usually small with respect to the number of variables, and it is crucial to adopt sound methodologies and strict experimental protocols to ensure statistical robustness [20]. Cross validation loops are valid approaches to avoid selection bias [21] and to separate training and test phases. The standard categorization proposed by Blum *et al*. [22] groups variable selection techniques in three main classes: filters, wrappers and embedded. Filters [23-25] are mostly based on ranking criteria where the features are ordered and then selected or discarded according to a fixed threshold. These methods are broadly employed due to their simplicity and fast computation, despite the lack of guarantee that the selection is optimal with respect to the class discrimination. In wrapper methods [26-28] the relevance of a feature subset is determined according to prediction performance of the learning algorithm itself, though variable selection and training are two separate processes. In contrast, embedded methods [29-34] have the advantage of incorporating feature selection within the construction of the classifier or regression model, i.e. as part of the training phase. We applied the embedded feature selection technique *l*_1_-*l*_2 _regularization with double optimization to the analysis of gene expression profile. This technique is based on the optimization presented by Zou *et al*. [35]. Theoretical studies [36] and empirical experiments [37,38] showed that such technique fulfills all the desirable properties of a variable selection algorithm. Indeed, the use of regularization allows performing embedded feature selection in the supervised learning framework, since the particular type of penalty used in *l*_1_-*l*_2 _regularization forces the classifier or the regression model to depend on a small number of selected features. Another asset of *l*_1_-*l*_2 _regularization is that it is multivariate by design since its solution is a classification or regression model that takes into account the combined effect of multiple features, and the set of relevant features is selected while looking at all the features at the same time. A strong advantage of *l*_1_-*l*_2 _regularization over other embedded methods is also its ability to take into account correlation among variables. In other words, when one variable is considered relevant to the problem, its correlated variables are considered relevant as well. While most feature selection techniques are based on heuristics, *l*_1_-*l*_2 _regularization is asymptotically consistent from the statistical viewpoint, i.e. theoretical results [36] guarantee that the best possible estimator is found as the number of training samples increases. Finally, the use of the double optimization allows to identify the relevant genes and to provide accurate discrimination. This approach was successfully applied in different contexts ranging from computer vision [37] to computational biology [38].

In this study we demonstrate that the application of *l*_1_-*l*_2 _regularization allows to model the effect of low oxygen tension, which was not detectable by supervised approaches, and to find a cluster of genes discriminating the normoxic and the hypoxic statuses of neuroblastoma cell lines.

## Results

### Experimental model

**Figure 1:**
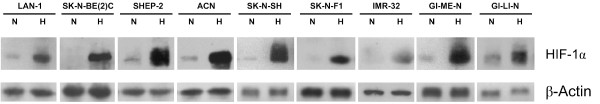
**Induction of HIF-1α by hypoxia in NB cell lines**. Western blot analysis of HIF-1α levels at normoxia (N) and hypoxia (H) in the 9 cell lines listed on top of the blot. Cells were cultured under normoxic or hypoxic (1%O_2_) condition for 18 hrs. Total protein lysates were analyzed by western blot using a mAb specific for human HIF-1α. A protein marker was run as a molecular-sized standard. The blot was rehybridized with anti-β-actin mAb to control for protein loading.

We generated an experimental model consisting of 9 different neuroblastoma cell lines that were cultured in a normoxic or hypoxic environment for 18 hrs. Table 1 shows the characteristics of the cell lines used. Each cell line was derived from a different patient and displayed a somewhat different phenotype. Four out of nine lines had MYCN amplification according to the literature [15]. We tested each cell line for MYCN mRNA expression and we found association between MYCN amplification and expression with the exception of SK-N-SH cell line in which there was expression without amplification (Table 1). To establish whether each cell line was sensitive to hypoxia, we measured by western blot analysis the induction of HIF-1α protein, a reliable indicator of cell exposed to low oxygen tension. The results (Figure 1) demonstrate that every cell line responded to hypoxia with a strong induction of HIF-1α protein, providing the biological validation of the model system. RNA was then extracted, processed and the gene expression profile was determined using the Affymetrix HG-U133 Plus 2.0 GeneChips. Thus, the dataset is represented by a n × p matrix, where n = 18 is the number of samples represented by normoxic or hypoxic neuroblastoma cell lines and p = 54613 is the number of probesets of the Affymetrix GeneChip.

**Table 1:** NB cell lines used and the relative characteristics

**Cell line**		**MYCN**		**Patient^a)^**		**Tumor characteristics^a)^**	
			
**name**	**Morphology^b)^**	**amplification**	**Expression^c)^**	**Age^d)^**	**sex**	**primary site**	**metastatic site^e)^**
			
ACN	neuroblast (N)	-	-	3.3	M	abdomen	BM, bone
SHEP-2	epithelial (S)	-	-	4	F	thorax	BM
GI-ME-N	neuroblast (N)	-	-	2	F	adrenal	LN, BM
SK-N-F1	epithelial (S)	-	-	11	M	unknown	BM
SK-N-SH	neuroblast/epithelial (I)	-	+	4	F	thorax	BM
SK-N-BE(2)c	neuroblast/epithelial (I)	+	+	2.2	M	unknown	BM
IMR-32	neuroblast (N)	+	+	1.1	M	abdomen	unknown
LAN-1	neuroblast (N)	+	+	2	M	unknown	BM, bone, LN
GI-LI-N	neuroblast (N)	+	+	1.11	F	adrenal	BM

^a)^For references on specific items see [15]. ^b)^N = neuroblastic; S = substrate adherant; I = intermediate. ^c)^Measured by northern blotting. (-) = below detection; (+) = higly expressed (see materials and methods). ^d)^Expressed as years.month. ^e)^BM = bone marrow; LN = lymphnode

### Unsupervised clustering analysis

**Figure 2:**
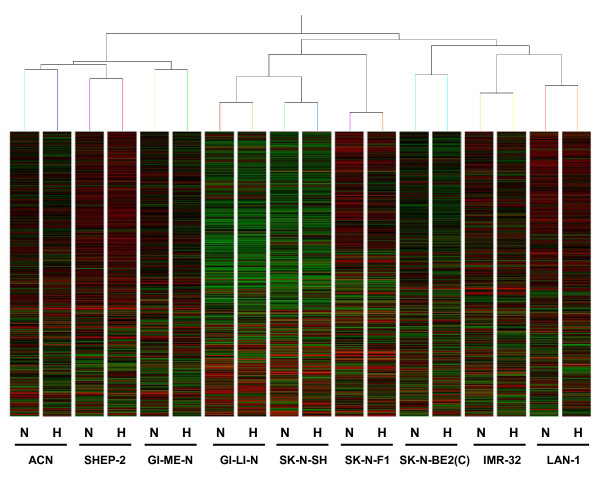
**Hierarchical clustering dendrogram**. Hierarchical clustering analysis of the 18 samples, listed at the bottom of the figure, representing the 9 cell lines in normoxic (N) or hypoxic (H) conditions using gene expression data of 54613 probesets. We used the correlation distance as similarity measure and complete linkage as cluster distance. Lines represent the probesets' expression and columns represent the samples. The correlation is shown above the dendrogram.

**Figure 3:**
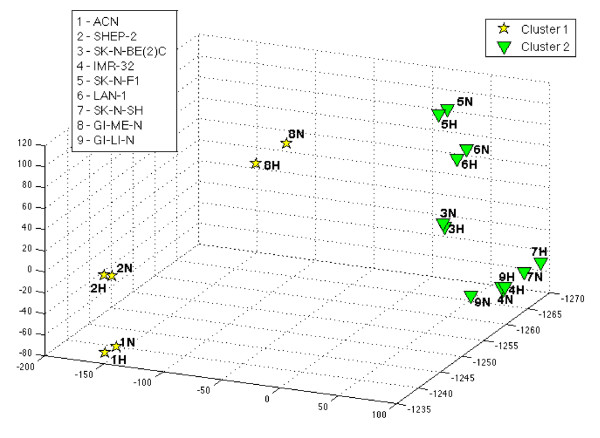
**3D Visualization of unsupervised analysis**. 3D projection via principal component analysis (PCA) of the clusters obtained with three clustering techniques (K-means, hierarchical clustering, and spectral clustering) applied to the 18 samples representing the 9 cell lines in normoxic (N) or hypoxic (H) conditions using gene expression data of 54613 probesets. The cell lines belonging to the cluster 1 (ACN N/H, SHEP-2 N/H, and GI-ME-N N/H) are represented by yellow stars while the cell lines belonging to the cluster 2 (SK-N-BE(2)C N/H, IMR-32 N/H, SK-N-F1 N/H, LAN-1 N/H, and SK-N-SH N/H) are represented by green triangles.

The purpose of our analysis was to determine the hypoxia signature by utilizing a strategy based on discriminative rules to detect the hypoxic status that does not depend on the specific cell line. We applied unsupervised analysis to the data set in order to determine whether the clustering discriminated between normoxic and hypoxic status. We first used hierarchical clustering with correlation distance as similarity measure (complete linkage). The dendrogram (Figure 2) shows that the cell lines cluster into two main groups. One cluster comprises ACN N/H, SHEP-2 N/H, and GI-ME-N N/H, whereas the second comprises SK-N-BE(2)C N/H, IMR-32 N/H, SK-N-F1 N/H, LAN-1 N/H, and SK-N-SH N/H cell lines. The hierarchical clustering demonstrated the existence of at least two groups of cell lines but did not separate the hypoxic from the normoxic transcriptome. Each cell line in normoxic status pairs with the corresponding hypoxic one because the distance between the two statuses of the same cell line is smaller than that between cell lines. We tested whether other unsupervised analysis techniques could distinguish the hypoxic status. We used spectral clustering and k-means techniques that may have a different performance. We found that the pattern of results was exactly the same across the various tests and clustered the cell lines, but not the hypoxic status, into two groups as it can be seen in Figure 3 where we projected each cell line on the three directions defined by Principal Component Analysis (PCA), and visualized them in the corresponding 3D-space. Clustering techniques are not endowed with a natural statistical score to asses the significance of the results and the reliability of the test is based on the comparison of the results obtained with different clustering techniques. The absolute concordance that we observed using three techniques argues for a good reliability of the results. We conclude that hypoxia unrelated responses associated with the nature of the cell lines mask the changes in gene expression associated with the transition to a hypoxic status. Visual inspection of the characteristics of the cell lines depicted in Table 1 indicated that MYCN expression/amplification could be one factor dichotomizing the cell lines. Major transcriptional changes in response to genes of the MYC family were described [39]. The highest correlation (correlation index of 0.89) was found between the obtained clusters and MYCN expression. SK-N-F1 represents the only exception because it does not express MYCN but it clusters with the MYCN positive cell lines.

In conclusion, the unsupervised approaches detected major transcriptome differences among the cell lines driven in part by the cascade of events triggered by MYCN expression. However, the disturbance generated by this transcriptional pattern was such that the detection of more subtle changes induced by hypoxia was completely masked.

### Supervised univariate analysis hypothesis test

**Figure 4:**
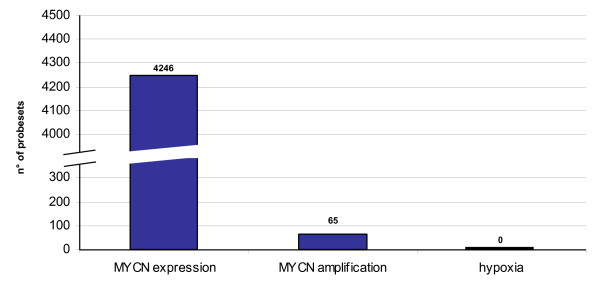
**T-test analysis**. Number of probesets differentially expressed (Y-axis) according to the t-test analysis with Benjamini-Hochberg multiple testing correction (p-value < 0.01). The analysis was performed searching for differentially expressed probesets between MYCN expressing vs. MYCN not expressing cell lines (MYCN expression); MYCN amplified vs. MYCN not amplified cell lines (MYCN amplification); normoxic vs. hypoxic cell lines (hypoxia).

In order to identify the hypoxia signature of the neuroblastoma cell lines, we attempted the classic approach of searching for probesets having different expression levels in the cells following exposure to low oxygen. We applied a t-test analysis with Benjamini-Hochberg correction [40] for multiple testing (p-value < 0.01). However, we did not identify any differentially expressed probesets between the two groups (Figure 4). Since the clusters identified by the unsupervised procedures are highly correlated with MYCN expression, we also applied a t-test analysis when the cell lines are divided into two classes based on MYCN expression (see Table 1). We found 4246 differentially expressed probesets comparing MYCN positive and negative cell samples and 65 differentially expressed probesets comparing normal and MYCN amplified samples (Figure 4). We conclude that the differential gene expression associated with hypoxia can not be brought out from the noise of other signals such as MYCN, with a classic supervised approach.

### Supervised multivariate *l*_1_-*l*_2 _regularization analysis

**Figure 5:**
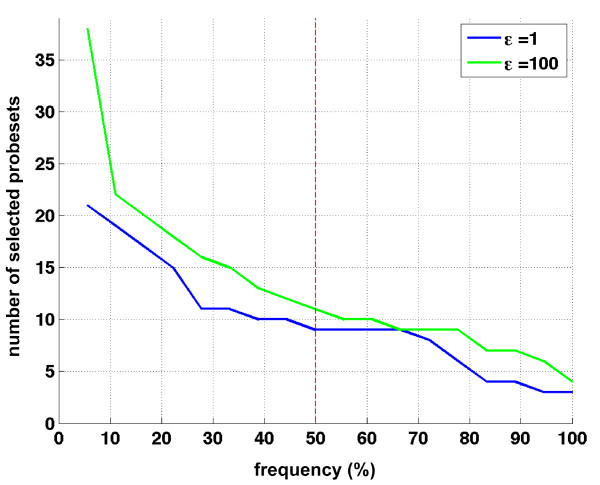
**Relative frequency in the 18 lists of the cross-validation loop**. Relative frequency of the selected probesets for ε = 1 and ε = 100 when *l*_1_-*l*_2 _regularization is applied to the 54613 probesets. The blue line indicates ε = 1 (minimal list). The green line indicates ε = 100 (correlation aware list). The graph shows the number of probesets selected by the algorithm for the two ε (Y-axis) at increasing frequency (X-axis). When we consider a threshold on the frequency at 50% we select 11 probesets with ε = 100 and 9 with ε = 1 (vertical red dashed line).

The impossibility to obtain a robust hypoxia signature by the previously described approaches prompted us to consider different algorithms based on a robust supervised variable selection technique, capable of detecting the hypoxia-induced transcriptome even in the presence of the disturbance of a strong competing signal. Toward this aim, we utilized the *l*_1_-*l*_2 _regularization algorithm according to the experimental protocol previously described [41]. The output depends on one free parameter ε that governs the amount of correlation allowed among the selected variables; the higher the ε, the more probesets are taken into account. We worked at the definition of a signature analyzing simultaneously all the probesets on the chip, thereby dealing with 54613-dim vectors. The system is characterized by a leave-one-out error of 3 out of 18 (17%) and it performed the validation loop producing 18 lists for each ε value. A common list was obtained as the union of the 18 lists, with a frequency score counting how many times each probeset was selected by the algorithm in the 18 cross validation loops. The results are shown in Figure 5, where the number of selected probesets is plotted against their frequency, for two values, 1 and 100, of the correlation parameter ε. The algorithm was able to identify a list of probesets that discriminated normoxic and hypoxic neuroblastoma cell lines despite the aforementioned disturbance in gene expression. Depending on the frequency score, the algorithm defined signatures comprising a number of probesets ranging from a maximum of 38 to a minimum of 3. The definition of one consensus hypoxia signature can be obtained setting the frequency threshold based on the behavior of each ε curve. The minimal list is obtained for values of ε equal to or lower than 1, whereas the largest list, which is correlation aware, is obtained for ε equal to 100. Due to the noisy nature of the dataset the system produced many unstable probesets whose relative frequency was lower than 30%. By observing the frequency curves in Figure 5, it can be noted that a plateau is present between 30% and 70% and we set the frequency threshold at the intermediate frequency of 50% (9/18). We set ε equal to 100 because we wanted to include every probesets concurring in the identification of the hypoxia status. The resultant consensus hypoxia signature is composed by the 11 probesets shown in Table 2 where they are sorted according to their selection frequency. These probesets represent 8 well characterized genes related to angiogenesis, apoptosis, glycolysis, and metabolism that are known to be induced by hypoxia in cells of different lineage (see references in Table 2). W57613 transcript, whose function is still unclear, was not previously known to be inducible by hypoxia.

**Table 2:** Hypoxia signature

**probeset^1)^**	**Gene Name**	**GenBank^2)^**	**f%^3)^**	**Description**	**References^4)^**
201848_s_at	BNIP3	U15174	100	BCL2/adenovirus E1B 19 kDa interacting protein 3	[49]
202887_s_at	DDIT4	NM_019058	100	DNA-damage-inducible transcript 4	[49]
226452_at	PDK1	AU146532	100	pyruvate dehydrogenase kinase; isoenzyme 1	[63]
236180_at	-	W57613	100	Transcribed locus, hypothetical protein FLJ11267	-
223193_x_at	E2IG5	AF201944	94	growth and transformation-dependent protein	[50]
225342_at	AK3L1	AK026966	94	adenylate kinase 3-like 1	[4]
224345_x_at	E2IG5	AF107495	89	growth and transformation-dependent protein	[50]
202022_at	ALDOC	NM_005165	78	aldolase C; fructose-bisphosphate	[63]
210512_s_at	VEGF	AF022375	78	vascular endothelial growth factor	[4,63]
201849_at	BNIP3	NM_004052	61	BCL2/adenovirus E1B 19 kDa interacting protein 3	[49]
235850_at	WDR5B	BF434228	50	WD repeat domain 5B	[64]

^a)^Probesets selected for frequency = 50% and ε = 100. ^b)^GenBank accession number. ^c)^Selection frequency in leave-one-out cross-validation. ^d)^Representative references describing the induction of the correspondent gene by hypoxia

**Figure 6:**
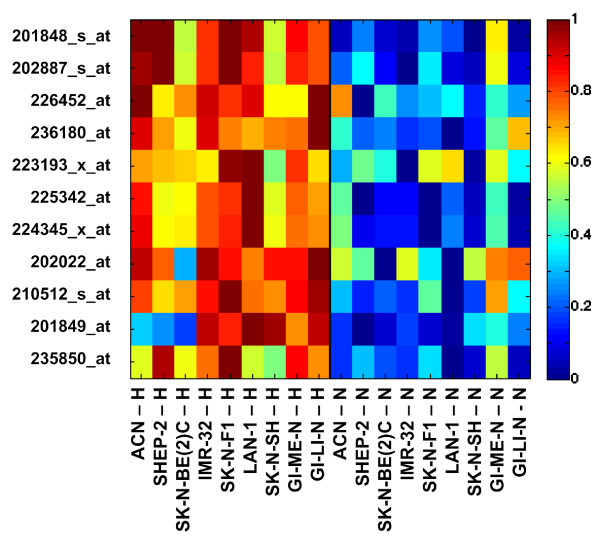
**Heatmap for the ε = 100 signature with frequency cut at 50%**. Normalized expressions for the 11 selected probesets in the 18 samples, listed at the bottom of the figure, representing the 9 cell lines in normoxic (N) or hypoxic (H) conditions. Red hues correspond to high expression, while blue indicates low expression values.

**Figure 7:**
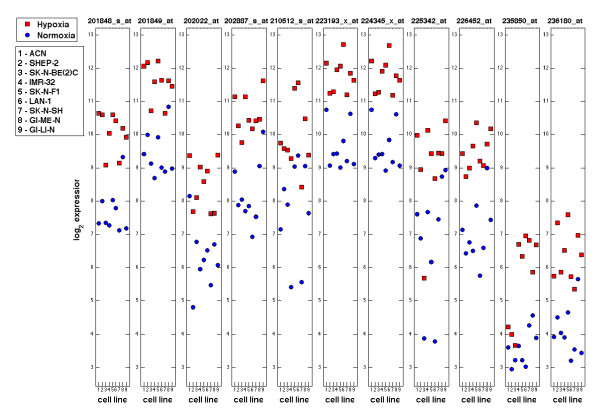
**Univariate representation of the cell lines based on the correlation aware list**. The graph shows the log-scale expression (Y-axis) for each of the 11 probesets selected in the signature measured on the NB cell lines (X-axis). The red square represents the cell line in hypoxic status, whereas the blue circle indicates the cell line in normoxic status.

**Figure 8:**
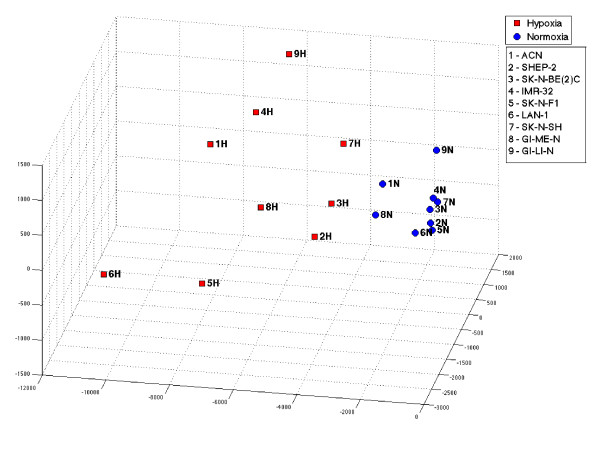
**3D projection of the cell lines**. This figure illustrates a 3-dimensional visualization of the data set restricted to the 11 selected probesets. The 3D representation is obtained by projecting the data submatrix onto its 3 principal components i.e. the components of maximum variance. Red squares, from 1H to 9H, represent the cell lines in hypoxic status and the blue circles, from 1N to 9N the corresponding cell lines in normoxia.

The expression levels of the selected probesets in the 18 samples are represented as a heatmap (Figure 6) which show a unequivocal partition of expression between normoxic and hypoxic cell lines. The actual levels of expression of the 11 probesets in the hypoxic and normoxic samples are shown in Figure 7 as a univariate representation in the log-scale expression. Although the normoxic and hypoxia statuses of each cell line are separated by the probesets expression, the gap is not equally large for all probesets and some overlapping in the selected cell lines is noticeable. The observation that, by projecting on the single probeset, the two statuses are only approximately separated may be attributed to the heterogeneity of the response of cell lines to hypoxia. The latter would cause differential modulation of probesets in the various cell lines, and individual probesets may not be perfectly split between the two statuses. However, these considerations do not impact on the strength of the consensus hypoxia signature that owes its robustness to its multivariate nature. The strong discriminative power of the consensus signature by a multivariate representation of the 11 probesets is shown in Figure 8. In order to obtain a 3D representation, the data submatrix is projected on its 3 principal components, i.e. the components of maximum variance. It is evident that two classes of normoxic and hypoxic statuses are clearly separated in the multidimensional space. This is due to the fact that *l*_1_-*l*_2 _regularization produces a multi-gene model and only the multidimensional representation can correctly visualize its strong discriminative power.

We conclude that *l*_1_-*l*_2 _regularization algorithm was able to identify 11 stable probesets that clearly separated the hypoxic from normoxic cell lines even in the case of the disturbance generated by the genetic alterations of the cell lines. Therefore, this cluster represents the consensus hypoxia signature hidden in the neuroblastoma cells transcriptome that we wanted to sort out.

Finally, we tested the ability of our signature to discriminate the hypoxic status in an out-of-sample schema. We considered two public datasets consisting of the gene expression profiles of primary cultures of immature dendritic cells [6] and of human astrocytes in response to hypoxia [42]. In both cases, we restricted the expression matrices to the 11 probesets and then applied regularized least squares in a leave-one-out cross validation loop, estimating the corresponding generalization error (see Table 3). In the astrocytes dataset, we assessed a cross validation error of 17%, comparable to that of the neuroblastoma cell lines. Gene Set Enrichment Analysis (GSEA) of our 11 probesets against neuroblastoma and astrocytes datasets also showed a significant enrichment in the hypoxic phenotype. In contrast, our 11 probesets signature showed a higher cross validation error when applied to dendritic cells (33%) and was not significantly enriched in the hypoxic phenotype (Table 3). These results indicate that our signature can be applied successfully to hypoxic systems, other than neuroblastoma, depending on the lineage/differentiation of the responding cell type.

**Table 3:** Out-of-sample validation

	**Cross validation**	**GSEA analysis**		
		
**Cell type**	**error^a)^**	**NES^b)^**	**p-Value^c)^**	**FDR^d)^**
neuroblastoma	17%	1.89	<0.01	0
astrocyte	17%	1.60	<0.01	0.045
dendritic cell	33%	1.19	0.21	0.869

^a)^Leave-one-out cross validation error estimated with regularized least squares. ^b)^GSEA normalized enrichment score for the hypoxia signature gene set. ^c)^Statistical significance of the enrichment score for the gene set. ^d)^q-Value of the false discovery rate. Values < 0.25 are considered acceptable.

## Discussion and Conclusion

We have analyzed the gene expression profile of 9 cell lines cultured in a normoxic or hypoxic environment in order to identify the hypoxia signature. We demonstrated that, differently from unsupervised approaches, *l*_1_-*l*_2 _regularization with double optimization identified a cluster of 11 stable probesets separating hypoxic from normoxic cell lines even when hidden in the neuroblastoma cells transcriptome characterized by the high disturbance of genetic alterations. Biological signatures can be derived from cell lines based datasets using many different informatics approaches (for review see [19]). This is the first report describing the use of the *l*_1_-*l*_2 _regularization with double optimization protocol described in [38] to distinguish datasets based on the biological status of the cells.

The first attempts to identify the hypoxia signature relied on three different unsupervised clustering analyses. These approaches detected major differences among the transcriptome of the cell lines driven by the characteristics of the cell lines themselves of which the cascade of events triggered by MYCN expression was a major component. However, the disturbance generated by these transcriptional patterns was such that the detection of more subtle changes induced by hypoxia was completely masked. This conclusion is supported by the results obtained with the supervised univariate analysis t-test which was able to identify a strong response associated to MYCN expression and, to a lesser extent to MYCN amplification, but not the hypoxia dependent response. The heterogeneity of neuroblastoma and neuroblastoma cell lines has been previously observed [43].

The impossibility to obtain a hypoxia signature by unsupervised approaches prompted us to consider different algorithms based on a robust supervised variable selection technique, capable of detecting the hypoxia-induced transcriptome even in the presence of the disturbance of a strong competing signal. The *l*_1_-*l*_2 _regularization allowed us to build a powerful discriminative rule and to define a signature of probesets also taking into account the presence of variables (probesets) correlated (collinear) with each other. The use of cross validation allows the selection protocol to generate an unbiased and objective output [21] beyond the theoretical results that guarantee the robustness of the core algorithm [36]. The strong discriminative power is proven by the 17% classification error that is a very low value when dealing with 18 samples and nearly 50 thousands variables.

We adopted a validation framework based on a double loop of leave-one-out cross-validation in order to extract unbiased estimates of the classification error. The outer loop produces 18 lists of relevant variables, from which we extract a common list by setting a frequency threshold. It can be appreciated from visual inspection of the frequency distribution that, for values lower than 30%, a large number of probesets is included, which are extremely unstable. For frequency above 70%, the number of selected probesets slowly decreases, and a plateau is present between 30 and 70%. Therefore, we set our frequency threshold to 50% that is the intermediate value of such a frequency plateau. The correlation parameter ε can be potentially tuned between 0 and +∞ in order to extract lists of probesets with different correlation degree. However, values of ε equal to or smaller than 1 provide the same minimal list which comprises 9 probesets. This list is minimal in that it does not include correlated probesets, and it can be viewed as the smallest set of variables needed to predict the hypoxic status without any prior information. Conversely, by increasing the correlation parameter ε we are able to expand the list to 11 probesets, which is obtained for ε ≥ 100. Since we are interested in all genes involved in the hypoxic condition, we define such a correlation-aware list as our hypoxia signature in neuroblastoma cell lines. This signature identifies also hypoxic status in astrocyte cell lines. We estimated a similar low cross validation error demonstrating that its application goes beyond the neuroblastoma lineage and providing an additional proof of its discriminatory power on out-of-set data. However, the neuroblastoma hypoxia signature is less efficient in discriminating hypoxic dendritic cells indicating the existence of a limited spectrum of hypoxic cell types that can be identified by this signature. Dendritic cells are terminally differentiated mononuclear phagocytes, biologically very far from the other cell types, deriving from hematopoietic precursors, short lived and programmed to serve as immunomodulatory cells [44]. This conclusion supports the concept of the heterogeneity of the response to hypoxia in different cell types [13,42,43]. The enrichment of the 11 probeset signature in the hypoxic phenotype of neuroblastoma cells and astrocytes provides biological validation of our approach by establishing a clear link between our signature and the hypoxic microenvironment.

The 11 probesets represents 8 genes all of which are known from the literature to be modulated by hypoxia in different cell types and to be part of key biological processes associated with the response to hypoxia, indicating, once more, the biological roots of our signature. ALDOC and PDK1 belong to the glycolytic pathway and are known to be up regulated by hypoxia in neuroblastoma [45]. Potentiating of the oxygen-independent glycolytic pathway to comply with the energy demand, is one of the major cellular response to hypoxia [46]. The energetic balance in cell metabolism has to be controlled by different mechanisms. For example, AK3L1 is known to be modulated by hypoxia and catalyzes the interconversion of adenine nucleotides playing an important role in cellular energy homeostasis in mitochondria [47]. DDIT4, induced by hypoxic stimulus, is an inhibitor of the mTOR signalling pathway, that results in inhibition of protein synthesis which, in turn, may affect the cellular tolerance to hypoxia by promoting energy homeostasis [48]. VEGF, a direct target of HIF-1, is secreted by a large variety of different hypoxic cells and promotes angiogenesis thereby favouring tumor growth and metastasis [4]. BNIP3 and E2IG5 are two genes promoting hypoxia-induced apoptosis observed mainly at very low oxygen concentrations [49]. E2IG5 is localized to mitochondria and facilitates apoptotic cell death via permeability transition, cytochrome c release, and caspase 9 activation [50]. Hypoxia is also known to increase histone H3 methylation through histone methyltransferase G9a [51]. WDR5B encodes for a protein that is the core component of histone methylation complexes, which are essential for histone H3 methylation. Thus, hypoxia might regulate chromatin organization and gene transcription by modulating WDR5B. Finally, the GenBank entry W57613 is part of the signature it is associated with a transcribed hypothetical protein FLJ11267 and was not previously known to be induced by hypoxia.

The novelty of our work is to introduce a rigorous and robust feature selection technique that can be exported to other experimental models and that is able to identify discriminative genes even in an adverse setting where the cell lines express great heterogeneity. The hypoxia signatures present in the literature show different sizes and composition [4,42,52-54]. The MSigDB [55] represents a valuable source of gene sets associated to the response to hypoxia. A first attempt to discuss our 11 selected probesets within the contest of 9 hypoxia signatures contained in the MSigDB is based on the analysis of the overlap among signatures (Table 4). One limitation of this comparison is that it must be based on gene names rather than probesets, because of the heterogeneity of the platforms. All the genes of our hypoxia signature, but one, are represented at least once in the 9 signatures. DDIT4 is the only hypoxia inducible gene that is included only in our signature. This comparison lends further support to the conclusion that the *l*_1_-*l*_2 _algorithm selected biologically relevant genes that have been included in other hypoxia signatures. There are at least three major reasons for the variability among the hypoxia signatures. The first is the diversity of the cell types as shown by Chi *et al*. [13] and Mense *et al*. [42] and supported by the observations on the heterogeneity among neuroblastoma cell lines by Fredlund *et al*. [43] and ourselves in this paper. Each cell responds to hypoxia on the bases of its own genetic make up, epigenetic constrains and differentiation stage. In fact, we show that our signature does not apply to dendritic cells, that are biologically very different from astrocytes and neuroblastoma. The second reason is the difference in the experimental setting and gene expression platforms. The need to collapse the microarray probes to gene names for comparisons is a direct consequence of this problem. The third, and more important issue, is the criterion used for assembling the signature. The majority of the signatures described so far, are based upon the representation of hypoxia associated biochemical pathways or the inclusion of differentially expressed genes rather than the essentiality and the discriminating power that we have chosen. Having defined the hypoxia signature as the minimal number of probesets capable of distinguishing normoxic and hypoxic gene expression profiles, our list is relatively short, not specific for a biochemical pathway, not relying on prior biological knowledge, but endowed with high discriminating power.

**Table 4:** Hypoxia gene signatures overlapping

**Gene Name**	**Overlap frequency^a)^**
DDIT4	0/9
PDK1	1/9
WDR5B	1/9
AK3L1	2/9
E2IG5	2/9
ALDOC	3/9
VEGF	4/9
BNIP3	5/9

^a)^Frequency of appearance of the genes in the 9 hypoxia signatures obtained from MsigDB (HYPOXIA_RCC_UP, MANALO_HYPOXIA_UP, MENSE_HYPOXIA_APOPTOSIS_GENES, HYPOXIA_FIBRO_UP, MENSE_HYPOXIA_TRANSPORTER_GENES, HYPOXIA_RCC_NOVHL_UP, HYPOXIA_REVIEW, MENSE_HYPOXIA_UP, HYPOXIA_NORMAL_UP).

The classification performance of our signature is evident in representations that indicate as first approximation the up-regulation of the signature in hypoxic condition. However, the multidimensional visualization is needed to fully appreciate the strong discriminative power of our signature because it takes into account its multivariate nature. In fact, when projecting over the individual probesets of the signature, the two classes are only approximately separated, since they appear either partially overlapping or very close. Indeed, since the *l*_1_-*l*_2 _regularization is a multivariate method, there is no need to expect a single probeset to have perfect discriminatory power on the classes, but one has to take into account the 11-dimensional model. While the normoxic cell lines are highly grouped and close to low expression values, the hypoxic lines are well spread over the multidimensional space, though well separated by the normoxic ones. Again, this behavior can be detected only by means of a multivariate analysis, since the analysis of individually regulated genes allows detecting only those probesets which multidimensional representation would see the hypoxic cell lines very well lumped together.

The advances in genome biology provide a growing and impressive amount of data. The challenge is to unmask specific, biologically relevant gene clusters that may be hidden by the disturbance of changes in an overwhelming number of unrelated genes. Our study demonstrated that the *l*_1_-*l*_2 _regularization framework is able to discriminate between two statuses of a cell that, albeit biologically very different, does not elicit a modulation of gene expression comparable in magnitude to that induced, for example, by genetic alterations. This scenario mimics the situation occurring in the tumor mass in which the signal will be perceived by cell differing in their genetic makeup, differentiation and progression in the cell cycle. The strategy described here can be readily applied to the detection of the response to other environmental signals such as small metabolites or pH changes to allow the creation of a database of tissue environment related variables that will ultimately be a great asset in unraveling the biology of the tumor and the possibly the description of better prognostic signatures.

## Methods

### Cells and culture conditions

The human neuroblastoma cell lines GI-LI-N, ACN, GI-ME-N, IMR-32, LAN-1, SK-N-BE(2)C, SK-N-F1, and SK-N-SH were purchased from the Interlab Cell Line Collection and SHEP-2 was kindly provided by Dr. Schwab (Division of Tumour Genetics, German Cancer Research Centre, Heidelberg, Germany). The cell lines were cultured in RPMI 16140 (Euroclone Ltd., Celbio, Milan, Italy), supplemented with 10% heat-inactivated fetal bovine serum (Sigma, Milan Italy), 2 mmol/L L-glutamine, 10 mM Hepes, 100 units/mL penicillin, and 100 μg/mL streptomycin (Euroclone Ltd), at 37°C in a humidified incubator containing 20% O_2_, 5% CO_2_, and 75% N_2_. Hypoxic conditions (1% O_2_) were achieved by culturing the cells in an anaerobic workstation incubator (BUG BOX, Jouan, ALC International S.r.l., Cologno Monzese, Milano, Italy) flushed with a gas mixture containing 1% O_2_, 5% CO_2_, and balanced N_2 _at 37°C in a humidified atmosphere. Oxygen tension in the medium was measured with a portable, trace oxygen analyzer (Oxi 315i/set, WTW; VWR International, Milano, Italy).

### Western blotting

Western blot analysis was done as detailed in [56]. Briefly, total cell lysates (100 μg) were electrophoresed on a 8% SDS-PAGE and electroblotted to Immobilon-P nitrocellulose membranes (Millipore, Billerica, MA). Immunoblotting was done with anti-HIF-1α mouse monoclonal antibody (BD Biosciences, San Jose, CA). An anti-β-actin mAb (Sigma) was used as an internal control for loading. Detection was carried out by enhanced chemiluminescence (Pierce, Rockford, IL) with peroxidase-conjugated goat anti-mouse or anti-rabbit antibodies (Sigma). Quantitative assessment of band intensities was carried out with the VersaDoc Image Analyzer (Bio-Rad, Hercules, CA).

### RNA extraction and northern blotting

Total RNA was extracted from cell lines using Trizol (Invitrogen Life technologies, Irvine, CA) according to the manufacturer's instructions. RNA was resuspended in diethyl pyrocarbonate-treated H2O (DEPC water), the physical quality control of RNA integrity was carried out by electrophoresis using Agilent Bioanalyzer 2100 (Agilent Technologies Waldbronn, Germany) and quantified by NanoDrop (NanoDrop Technologies Wilmington, Delawere USA). 2 μg of total RNA from each sample were electrophoresed under denaturing conditions on a 1.2% agarose gel containing 2.2 mol/L formaldehyde and transferred to Nytran membranes. A RNA marker was run in parallel as a molecular-sized standard. Filter hybridization was done with 2 × 106 cpm/mL of 5'-[α32P]dCTP-labeled human MYCN cDNA, in Hybrisol I hybridization solution (Oncor, Gaithersburg, MD) as described previously [57]. Blots were autoradiographed with Kodak XAR-5 film (Eastman Kodak, Rochester, NY), and quantitative assessment of the band intensities was carried out with the VersaDoc Image Analyzer (Bio-Rad Laboratories, Hercules, CA).

### Microarray experiments

Total RNA from neuroblastoma cell lines in normoxic and hypoxic conditions was reverse transcribed into cDNA and biotin labeled according to the Affymetrix instructions (Affymetrix, SantaClara, CA). Biotin-labeled cRNA was cleaned up with the Qiagen RNeasy Mini kit and ethanol precipitation, checked for quality with Agilent Bioanalyzer 2100, and fragmented by incubation at 94°C for 35 min in 40 mmol/L Tris-acetate (pH 8.1), 100 mmol/L potassium acetate, and 30 mmol/L magnesium acetate. Fragmented cRNA was used for hybridization to Affymetrix HG-U133 Plus 2.0 arrays. GeneChips were scanned using an Affymetrix GeneChip Scanner 3000. All microarrays were examined for surface defects, grid placement, background intensity, housekeeping gene expression, and a 3':5' ratio of probe sets from genes of various lengths. Gene expressions were then extracted from CEL files and normalized using the Robust Multichip Average (RMA) method [58] by running a R script using the Bioconductor [59] package *affy *[60].

The complete data set for each microarray experiments (accession number GSE15583) was uploaded in the Gene Expression Omnibus public repository at National Center for Biotechnology Information.

### Unsupervised methods

We adopted three clustering methods, k-means, hierarchical clustering and spectral clustering [61]. K-means and hierarchical clustering require defining a similarity measure (or a distance) between points and a corresponding distance between a point and a cluster. K-means procedure follows a simple and easy way to classify a given data set through a certain number of clusters (assuming k clusters) fixed a priori. The main idea is to define k centroids, one for each cluster. These centroids should be placed in a cunning way because of different location causes different results. Hierarchical clustering proceeds by agglomerating into clusters points that are similar to each other or similar to a previously found group of points. The algorithm stops when all the points and the clusters collapse in a single cluster. The user is asked to decide when to stop the procedure hence defining the number of clusters to be found. The results of the clustering procedure can be visualized using the so called dendrogram. We used correlation distance as a similarity measure among points and complete linkage as clusters distance. Spectral clustering is based on the idea of recursively dividing the data into homogenous clusters. As the number of data increases the obtained clusters converge to an ideal/optimal clustering. The algorithm requires defining a similarity among the examples in the data set. Such a similarity function is used to build the so called Graph Laplacian. If we denote with W the n × n similarity matrix among the examples in the data set and D the diagonal matrix whose entries are the sum of the rows in W, then the Graph Laplacian is defined as L = I-W/D. This latter is a n × n matrix whose eigenvectors have special meaning. The second eigenvector in fact allows partitioning the data in two disjoint sets. Each entry of the vector is associated to an example. Examples corresponding to positive entries are assigned to a cluster and examples corresponding to negative entries are assigned to another cluster. More clusters are defined looking at the following eigenvectors (multiway spectral clustering) or recursively applying the procedure on each cluster separately. The eigenvectors of the Graph Laplacian has a further property. Similarly to principal components analysis (PCA) they can be used to perform dimensionality reduction. The corresponding procedure is known as Laplacian-eigenmaps. Differently to PCA where the data are projected on the directions of maximal variance, in Laplacian-eigenmaps the first eigenvectors entail the direction preserving the distance among the examples. This last property makes Laplacian-eigenmap an ideal tool for data visualization. In spectral clustering, for each sample we evaluate the average of the distances with its 5 nearest neighbors and select σ as the average over all the considered samples

### Supervised methods for gene selection - *l*_1_-*l*_2 _regularization

Our approach to feature selection is the *l*_1_-*l*_2 _regularization with double optimization described in [38]. The method is based on the optimization principle presented in [35] and further developed and studied in [36]. To illustrate such method we first fix some notation in the learning framework. Assume we are given a collection of n examples/subjects, each represented by a p-dimensional vector x of gene expressions. Each sample is associated with a binary label Y, assigning it to a class (e.g. patient or control). The dataset is therefore represented by a n × p matrix X, where p >> n and Y is the n-dimensional labels vector. We consider a linear model f(x) = ⟨x,β⟩. Note that β = β_1_,...,β_p _is a vector of weight coefficients and each gene is associated to one coefficient. A classification rule can be then defined taking sign(f(x)) = sign(⟨x,β⟩). If β is sparse, that is some of its entries are zero, then some genes will not contribute in building the estimator. The estimator defined by *l*_1_-*l*_2 _regularization solves the following optimization problem:



where the least square error is penalized with the *l*_1 _and *l*_2 _norm of the coefficient vector. The least square term ensures fitting of the data whereas adding the two penalties allows to avoid over-fitting. The relative weight of the two terms is controlled by the parameter ε. The role of the two penalties is different, the *l*_1 _term (sum of absolute values) enforces the solution to be sparse, the *l*_2 _term (sum of the squares) preserves correlation among the genes. This approach guarantees consistency of the estimator [36] and enforces the sparsity of the solution by the *l*_1_term, while preserving correlation among input variables with the *l*_2 _term. Differently to [35] we follow the approach proposed in [38], where the solution β_*l*1*l*2_, computed through the simple iterative soft-thresholding, is followed by a second optimization, namely regularized least squares (RLS), to estimate the classifier on the selected features. The parameter ε in the *l*_1_-*l*_2 _regularization is fixed a priori and governs the amount of correlation. By tuning ε we obtain a one-parameter family of solutions which are all equivalent in terms of prediction accuracy, but differ on the degree of correlation among the selected features. The training for selection and classification requires the choice of the regularization parameters for both *l*_1_-*l*_2 _regularization and RLS denoted with λ* and τ*, respectively. Hence, statistical significance and model selection is performed within double selection bias free cross validation loops (see [41] for details). In order to assess a common list of probesets, it is necessary to choose an appropriate criterion [62]. We based ours on the *frequency*, i.e. we decided to promote as relevant variables the most stable probesets across the lists. The complete validation framework comprising the *l*_1_-*l*_2 _regularization is implemented in MATLAB code (available at )

### Univariate analysis via hypotheses test

We test the hypothesis of equal distribution of the probesets in the two different statuses by means of t-statistic. We correct for multiple hypothesis testing with Benjamini and Hochberg method for controlling the False Discovery Rate [40].

### Out-of-sample analysis

To assess the generalization properties of the signature, we used the publicly available gene expression profile datasets of immature dendritic cells (GEO accession number: GSE6863) and astrocytes (GSE3045) cultured under normoxic and hypoxic conditions. Both datasets consist of 6 samples (3 hypoxic and 3 normoxic cell lines) and are measured on the Affymetrix HG-U133 Plus 2.0 GeneChip. The dendritic cells dataset was normalized with the RMA method, similarly to the neuroblastoma cell lines. We could not repeat the same procedure for the astrocytes data, since the .CEL files were not available. We therefore had to use the previously normalized intensities published on the Gene Expression Omnibus. As a measure of relevance of our signature we used its prediction accuracy on out-of-sample data. For each dataset, we restricted the expression matrix to a submatrix of the 11 variables of the inferred signature and we estimated the generalization error of a Regularized Least Squares classifier in a leave-one-out cross-validation loop.

Gene Set Enrichment Analysis (GSEA) [55] was used to determine if the members of our hypoxia gene signature were generally associated with hypoxic status, and was therefore performed on all probesets on the HG-U133 Plus 2.0 GeneChip. A normalized enrichment score (NES) was calculated for the gene set and the statistical significance of the NES was estimated by an empirical permutation test using 1.000 gene permutations to obtain the nominal p-value and a false discovery rate.

## Authors' contributions

PF and LV conceived the initial idea, the experimental design, supervised the work, and wrote the manuscript. PF performed microarray experiments. AB contributed with the development of MATLAB and R scripts for data processing, normalization and analysis, performed the t-test and supervised analysis, and visualized the results. SM wrote the core code for *l*_1_-*l*_2 _regularization and contributed with the development of MATLAB and R scripts for data processing, normalization analysis. LR performed the unsupervised analysis and helped in the design and implementation of the supervised analysis. AB, SM and LR contributed to the writing of the manuscript. AV supervised the entire statistical data analysis. All authors read and approved the manuscript.
